# Sanqi Panax Notoginseng Injection for Angina Pectoris

**DOI:** 10.1155/2014/963208

**Published:** 2014-02-16

**Authors:** Xiaochen Yang, Xingjiang Xiong, Jie Wang

**Affiliations:** Department of Cardiology, Guang'anmen Hospital, China Academy of Chinese Medical Sciences, Beixiange 5, Xicheng District, Beijing 100053, China

## Abstract

*Objective*. To evaluate the clinical curative effects of SPN injection on AP. *Methods*. Six databases were systematically searched including Cochrane Central, PubMed, EMBASE, CBM, Chinese National Knowledge Infrastructure (CNKI), and VIP to identify randomized controlled trials (RCTs). We assessed the quality of included studies, extracted valid data, and undertook meta-analysis following the steps of systematic review recommended by the Cochrane group. *Results*. Ten moderate-to-low quality randomized controlled trials involving 969 patients were included. There was no evidence that SPN alone had better or worse effects than conventional drugs on improving clinical symptoms (RR 1.09, 95% CI 0.85 to 1.39) and ECG (RR 1.17, 95% CI 0.84 to 1.63). However, there was an evidence that SPN combined with western medications was a better treatment option than conventional drugs alone in improving clinical symptoms (RR 1.28, 95% CI 1.19 to 1.33) and ECG (RR 1.27, 95% CI 1.12 to 1.45). No serious adverse effects were reported. *Conclusion*. Compared with conventional treatment, SPN may show the potential of optimizing symptomatic outcomes. As a kind of alternative and complementary medicine, SPN may provide another choice for AP patients and further large-scale high-quality trials are needed to confirm this efficacy.

## 1. Introduction

Ischemic heart disease (IHD) is characterized by an imbalance of the coronary blood flow and myocardial requirement [1], usually with symptomatic discomfort in the chest, that is, angina pectoris (AP) [2]. AP, especially unstable angina pectoris (UAP), may represent the first clinical manifestation of IHD, and it can follow an acute coronary syndrome (ACS) [3]. Meanwhile, IHD can lead to acute myocardial infarction (AMI) or fatal arrhythmia or cause a number of chronic sequelae, mostly prominently stable angina pectoris (SAP) or heart failure [4]. According to a survey of a community in Beijing, the incidence of AP and myocardial infarction is 30.7% (male 26.2%, female 33.7%) and 2.9% (male 4.8%, female 1.7%), respectively [5]. Conventional medicines for reducing ischemic symptoms and improving prognosis of angina pectoris include nitrates, beta blockers, calcium antagonists, aspirin, and ACE inhibitors. However, all these drugs can have undesirable effects. For example, the abuse of nitrates carries the risks of tolerance and rebound, and can cause headaches, flushed cheeks, and other adverse reactions [6].

In recent decades, with the growing and sustained interest in the benefits of herbal medicines, Sanqi (radix notoginseng) has caused much attention for its good cardiovascular effects. Sanqi consists of a mixture of compounds, among which Panax notoginseng saponins (PNS) representing the most biologically active ingredient [7]. About twenty-seven saponins were identified from PNS, most of these monomer components are 20(S)-protopanaxadiol and 20(S)-protopanaxatriol. Among them the content of Rg1 and Rb1 is higher, as the main saponins of radix notoginseng [8]. Animal and cellular studies have shown various potential benefits of the agent, including (1) inhibiting platelet aggregation and plasma coagulation [9], (2) protecting myocardium cells from apoptosis through activating PI3K/Akt signaling pathway [10], (3) promoting human umbilical vein endothelial cells (HUVEC) proliferation and secretion of vascular endothelial growth factor (VEGF) [11], (4) ameliorating anti-myocardial ischemia injuries by decreasing oxidative stress and repressing inflammatory cascade [12], (5) declining in serum levels of total cholesterol, triglycerides, and low density lipoprotein-cholesterol [13], (6) modulating Toll-like receptor (TLR) ligand-induced activation of cultured dendritic cells (DC) 2.4 cells [14], (7) inhibiting the vascular intimal hyperplasia, significantly lowering the expression of proliferating cell nuclear antigen (PCNA), cyclinE, cyclinD1, fibronect (FN) and matrix metalloproteinase-9 (MMP-9) [15], (8) suppressing phosphorylation of FAK on threonine 397, integrins expression, and NF-*κ*B translocation [16], and (9) inhibiting cell proliferation and reversing basilar artery remodeling [17].

In clinical practice, the available PNS agent, which is approved by State Food and Drug Administration of China, includes Sanqi Panax Notoginseng (SPN) injection for intravenous treatment and Xuesaitong soft capsule for oral treatment. PNS, which including notoginsenoside R1, ginsenoside Rb1, Rg1, are extracted from the raw root of Sanqi and then chemically derivatized into water-soluble PNS for the preparation of injection. The common dosage for administration of SPN is 200–400 mg per day. SPN injection is given diluted at the point of treatment in 250–500 mL 5% glucose injection for intravenous administration. It is widely used in Chinese hospitals for cardiovascular diseases.

This study aimed to systematically and objectively evaluate the clinical curative effect and safety of SPN for AP based on a general understanding of previous research and meta-analysis of randomized controlled trials (RCTs) on AP.

## 2. Methods

### 2.1. Search Strategy

We systematically searched for studies on SPN published without language restriction. The databases that were searched included PubMed, the Cochrane Center Controlled Trials Register (2013), EMBASE (1980–2013), Chinese National Knowledge Infrastructure (CNKI, 1979–November 2013), Chinese Biomedical Literature Database (CBM, 1978–November 2013), and Chinese Scientific Journal Database (VIP, 1989–November 2013) by two authors (X. Yang and X. Xiong). The English searching terms were used individually or combined including “angina pectoris”, “Sanqi Panax Notoginseng injection,” “randomized controlled trial”, “controlled clinical trial”, “randomly”, “trial”, “randomized,” and “randomized”. The Chinese searching terms were used individually or combined including those for the generic name of angina pectoris (*“Xian_jiao_tong”*), Sanqi Panax Notoginseng injection (*“Xue_sai_tong_zhu_she_ye”*), and randomized (*“sui_ ji”*).

### 2.2. Study Selection

Both the titles and abstracts of potentially relevant studies were identified through searching and reviewed independently by 2 reviewers (X. Yang, and X. Xiong). The randomized controlled trials that evaluated the cardiovascular effects of SPN for AP were included: (a) participants were suffering from and being treated for angina pectoris; (b) the study was claimed to be an RCT; (c) the study compared SPN with western medicine in efficacy; (d) duration of treatment was at least four weeks. Duplicated or redundant studies were excluded. If there were discrepancies in the process of selection, whether to include or exclude a study was resolved by a third author (J. Wang).

### 2.3. Inclusion Criteria of Interventions and Participants

The trials include intervention and comparison of any of the following: (1) SPN plus routine treatment versus routine treatment; (2) SPN plus routine treatment versus routine treatment + placebo; (3) SPN versus routine treatment. The participants who were suffering from and had been diagnosed as UA should be included regardless of the severity. The Diagnosis of AP [18] was according to “the International Society and Federation of Cardiology/World Health Organization (ISFC/WHO).”

### 2.4. Inclusion Criteria of Outcome Measures

The primary outcome measure was mortality due to ischemic heart disease or incidence of heart events, for instance, AMI, severity arrhythmia, heart failure, and revascularization. The secondary outcome measure was symptomatic and ECG improvements. Effective symptomatic improvements should achieve at least 50% (basic) or 80% (significant) reduction of angina symptoms (RAS). Effective ECG improvements should achieve at least 0.05 mV lowering at ST segment in ECG (basic) or nearly normal (significant) ECG during an exercise test as suggested in the ACC/AHA guideline [19]. Other outcomes like frequency of angina attack (FAA), duration of angina attack (DAA), follow-up, and adverse events were also measured.

### 2.5. Methodological Quality Assessment

Six specific domains were used to assess the qualities of included RCTs including random sequence generation, allocation concealment, blinding of participants and personnel, blinding of outcome data, incomplete outcome data, and selective reporting. Two authors (X. Xiong and X. Yang) independently assessed the methodological quality of included RCTs using RevMan 5.1.0. This judgment on the risk of bias is categorized by three levels: “high risk,” “unclear risk,” and “low risk.” Trials which met all criteria were judged as having a low risk of bias, trials which met none of the criteria were judged as having a high risk of bias, and trials with insufficient information to judge were classified as unclear risk of bias.

### 2.6. Statistical Analysis

Two authors (X. Yang and X. Xiong) used RevMan 5.1.0 provided by Cochrane Collaboration to perform a meta-analysis of RCTs if the intervention, control, and outcome were the same or similar [20]. Dichotomous data were expressed as risk ratio (RR) and continuous outcomes as weighted mean difference (WMD), with their 95% confidence intervals (CI), respectively. The statistical heterogeneity was examined with the *I* ^2^-test. In the absence of significant heterogeneity (*I* ^2^ < 50%), a fixed-effect model was used; otherwise random-effects model was used for pooling data (*I* ^2^ > 50%).

## 3. Result

**Figure 1:**
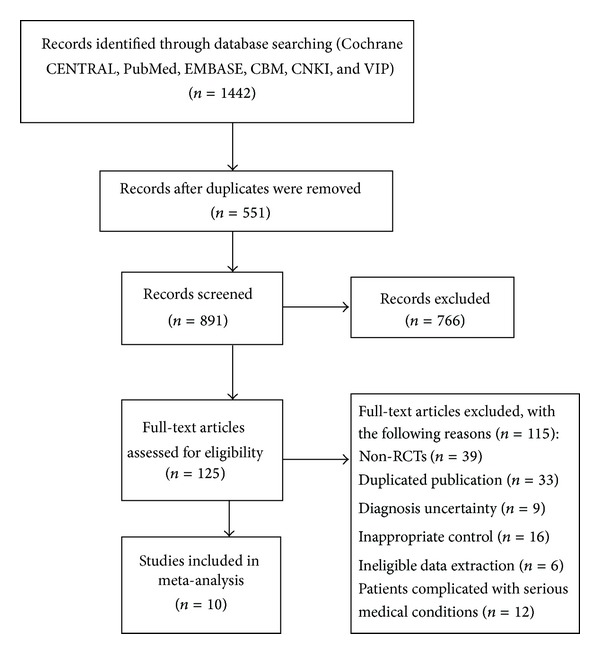
Flow chart of study search and selection.

A total of 1442 studies were identified through searching 6 electronic databases. After screening and examining the titles and abstracts of studies, 125 of them were included for further assessment. We excluded the duplicates, animal experiments, reviews, case reports, and so on for not meeting the inclusion criteria. The full texts of all 125 articles were reviewed carefully by two authors (X. Xiong and X. Yang) and other ineligible studies were excluded. Finally, 10 RCTs [21–30] were included which had been conducted and published in Chinese (see Figure 1).

### 3.1. Study Characteristics

The study characteristics of 10 RCTs were listed in Table 1. The 10 RCTs, which involved 969 patients, aged 18 to 83, were published between 2002 and 2011. Among 969 patients, 346 participants were UAP, 465 participants were SAP, and other 158 participants just mentioned with AP. Treatment courses ranged from 2 to 4 weeks. Only one study [25] reported follow-up data and adopted mortality as primary outcome. The daily dosages of SPN were 400 mg in four studies [21, 26, 29, 30] and 500 mg in the other six studies [22–25, 27, 28]. SPN injection was given diluted at the point of treatment in 20 mL 25% glucose injection for intramuscular administration or in 250–500 mL 5% glucose injection for intravenous administration. The symptomatic and ECG improvements were reported in all ten studies, while two studies [21, 27] also reported changes of frequency of angina attack and duration of angina attack, respectively. The other outcomes included blood lipid, C-reactive protein (CRP), and adverse effects. There were two comparisons: (I) SPN versus conventional medicines; (II) SPN plus conventional medicines versus conventional medicines.

### 3.2. Quality Assessment of the Included Studies

**Figure 2:**
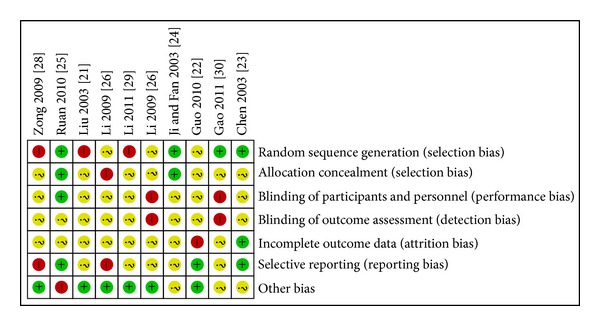
Risk of bias summary: review authors' judgments about each risk of bias item for each included study.

**Figure 3:**
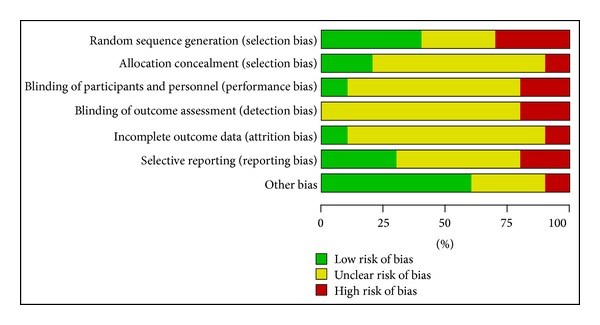
Risk of bias graph: review authors' judgments about each risk of bias item presented as percentages across all included studies.

Cochrane risk of bias tool was used to assess of risk bias. All 10 RCTs mentioned the word “randomization,” but none of them described it in details. Only one study [24] mentioned the performance of single blinding. None mentioned allocation concealment or intention-to-treat analysis. Because the protocols of all the 10 included trials were not accessible, selective reporting was generally unclear (Figures 2 and 3).

### 3.3. Efficacy and Safety Analysis

All 10 RCTs were divided into two subgroups for further analysis with consideration of clinical heterogeneity across the studies. Group I compared SPN plus conventional drugs with conventional drugs alone, while Group II evaluated the effects of SPN relative to conventional drugs. Descriptions and interpretations of angina and ECG improvement for patients in all studies followed the rules prescribed in the *Efficacy Criteria for Angina of Coronary Heart Disease* [31].

### 3.4. Primary Outcomes

One trial [25] adopted mortality as the primary outcome. After one year of follow-up, 1 case of AMI in SPN plus conventional drugs group, while 4 cases of AMI in conventional drugs and 6 cases of AMI in SPN group.

### 3.5. Subgroup Analysis of Secondary Outcomes

**Figure 4:**
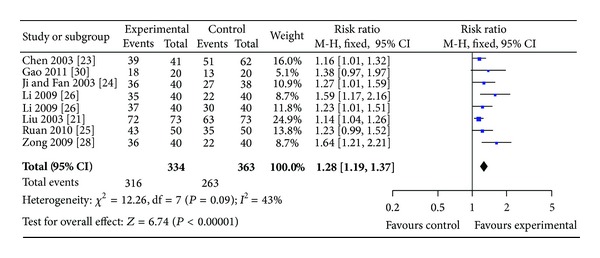
Forest plot of comparison: SPN plus conventional drugs versus conventional drugs; outcome: RAS.

**Figure 5:**
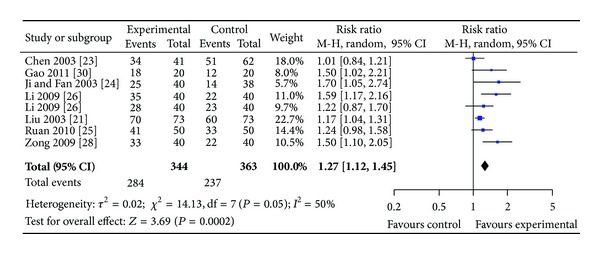
Forest plot of comparison: SPN plus conventional drugs versus conventional drugs; outcome: ECG.

*(i)  Group I. SPN Plus Conventional Drugs versus Conventional Drugs*. Eight studies involving 707 participants in the treatment group reported angina and ECG improvement. No statistical heterogeneity was found among these studies in angina improvement (*P* = 0.09 > 0.05, *I* ^2^ = 43%); however, heterogeneity was found in ECG improvement (*P* = 0.05, *I* ^2^ = 50%) (see Figures 4 and 5). The results showed that SPN plus conventional drugs achieved statistically significant improvement of clinical symptoms than conventional drugs alone (RR 1.28, 95% CI 1.19 to 1.33, Figure 4) and improvement of ECG (RR 1.27, 95% CI 1.12 to 1.45, Figure 5).

**Figure 6:**
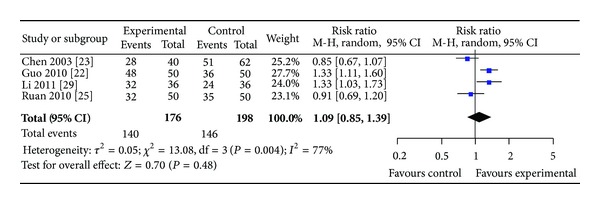
Forest plot of comparison: SPN versus conventional drugs; outcome: RAS.

**Figure 7:**
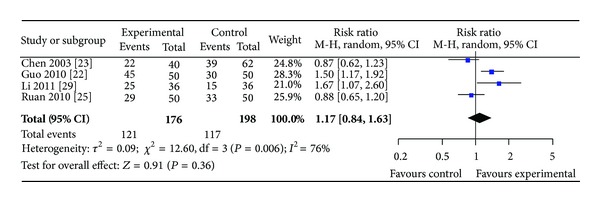
Forest plot of comparison: SPN versus conventional drugs; outcome: ECG.

*(ii)  Group II. SPN versus Conventional Drugs*. Four studies involving 374 participants in the treatment group reported angina and ECG improvement. Statistical heterogeneity was found among these studies in angina improvement (*P* = 0.004, *I* ^2^ = 77%) and in ECG improvement (*P* = 0.006, *I* ^2^ = 76%) (see Figures 6 and 7). The results showed that SPN achieved no significant improvement of clinical symptoms than conventional drugs alone (RR 1.09, 95% CI 0.85 to 1.39, Figure 6) and improvement of ECG (RR 1.17, 95% CI 0.84 to 1.63, Figure 7).

### 3.6. Other Outcomes (Frequency and Duration of Angina Attack, BL, and CRP)

Compared with conventional medicine, one trial [21] indicated that duration of angina attack decreased 8.9 ± 3.5 min to 6.5 ± 2.4 min favoring SPN plus conventional medicine. The other trial [27] indicated that frequency of angina attack decreased 4.21 ± 2.05 times to 2.47 ± 1.57 times favoring SPN plus conventional medicine. Four trials [22, 23, 26, 28] showed that, after treatment, the level of BL decreased significantly (*P* < 0.05) in SPN group compared to control group. Two trials [26, 30] showed that after treatment, the level of CRP decreased significantly (*P* < 0.05) in SPN plus conventional drugs group compared to control group.

### 3.7. Publication Bias

We failed to perform funnel plot to detect publication bias because of insufficient number of trials.

### 3.8. Adverse Effect

A total of seven trials (70%) [22–27, 29] reported adverse effects relating to the treatment by SPN combined with conventional drugs or used alone. The adverse effects only included 7 cases of headache 1.53% (7/456) and 3 cases of rash 0.65% (3/456). No severe adverse events were reported.

## 4. Discussion

According to our results, SPN appeared to be an effective and safe treatment option for the treatment of AP, and SPN plus conventional drugs appeared to be more effective than conventional drugs alone.

A total of 10 randomized trials including one with 969 participants were included in our review for AP. Improving patient outcomes is one of the primary goals of AP management. Several evidences have proved that traditional Chinese medicine (TCM) is a holistic system of medicine and have unique theories of the treatment, especially for relieving symptoms [32–34]. Compared with conventional drugs, SPN has the potential of being more effective on the improvement of clinical symptoms and ECG; however, these advantages have been obvious based on the low-level evidences collected so far. The results in Groups I and II show that SPN with or without routine conventional drugs could improve the degree of angina and reduce the frequency of angina attacks after 2–4 weeks of treatment. Besides these presenting symptoms of the patients, objective measurements such as ECG also provided evidence. However, several limitations should be considered before accepting the findings of this paper.

Although all 10 RCTs claimed that the positive effect of SPN combined with conventional drugs was better than conventional drugs alone. We still cannot make firm conclusions due to poor methodological quality. All trials reported randomization but lacked details on the method of randomization. They failed to provide enough information for judging whether the randomization procedures had been carried out properly. No multicenter, large-scale RCTs were identified. No dropouts and withdrawals were described. No placebo control was used and none of the trials were described as double blind. All of the conventional drugs therapies varied from trial to trial. It should also be pointed out that the different measurements of outcome were all short-term outcomes, such as symptoms relief before and after treatment. The durations of treatment were various, and reported methods were diverse in included trials. The long-term effects of SPN on patients with AP and its role in the prognosis were scarcely discussed.

None of the included trials reported severe adverse events possibly related to XST, and the adverse effects included 7 cases of headache 1.53% (7/456) and 3 cases of rash 0.65% (3/456) only. We cannot draw firm conclusions about the safety of XST since 3 of 10 trials did report information on safety.

Since all of the trials were of small size with positive results and were conducted in China, geographic biases may be induced. The 10 trials included in this review were of moderate-to-low quality. As a result, the evidences need to be interpreted with caution. While SPN is a widely used therapy for AP in China, the results of the present review suggest that high-quality controlled trials are required for assessing long-term safety by designing a longer duration of treatment and a long-term follow-up.

## 5. Conclusion

To the best of our knowledge, this review is the first to systematically evaluate the effects of SPN in the treatment of AP, addressing the lack of this type of research. In conclusion, SPN shows potential in treating AP; however, the findings should be interpreted with caution due to the low quality of included trials. Rigorous multicenter, large-scale clinical trials must be carried out to reveal the exact effectiveness in the future.

## Figures and Tables

**Table 1:** The characteristics of included RCTs of Sanqi Panax Notoginseng injection for AP.

Study ID	Sample	Type of angina	Diagnosis standard	Age	Intervention group	Control group	Course (week)	Outcome measures
Liu et al. 2003 [21]	146	UAP	1979 ISFC/WHO	46–74	SPN + conventional drugs	Conventional drugs	2	RAS, ECG, DAA
Guo 2010 [22]	100	SAP	1979 ISFC/WHO	40–82	SPN	Isosorbide mononitrate	2	RAS, ECG, BL, adverse event
Chen et al. 2004 [23]	143	SAP	1979 ISFC/WHO	45–79	SPN SPN + isosorbide mononitrate	Isosorbide mononitrate	3	RAS, ECG,, BL, adverse event,
Ji and Fan 2003 [24]	78	AP	GCRNDTCM	49–83	SPN + conventional drugs	Conventional drugs	2	RAS, ECG, adverse event
Ruan 2010 [25]	150	SAP	GCRNDTCM	44–71	SPN SPN + isosorbide mononitrate	Isosorbide mononitrate	2	RAS, ECG, adverse event Follow-up
Li 2009 [26]	80	UAP	GCRNDTCM	18–80	SPN + conventional drugs	Conventional drugs	4	RAS, ECG, BL, CRP, adverse event
Li 2009 [27]	80	UAP	1979 ISFC/WHO	46–74	SPN + conventional drugs	Conventional drugs	4	RAS, ECG, FAA, adverse event
Zong et al. 2009 [28]	80	AP	1979 ISFC/WHO	51 ± 11	SPN + conventional drugs	Conventional drugs	2	RAS, ECG, BL
Li et al. 2011 [29]	72	SAP	1979 ISFC/WHO	66 ± 8	SPN	Nitroglycerin	2	RAS, ECG, adverse event
Gao et al. 2011 [30]	40	UAP	1979 ISFC/WHO	52 ± 8	SPN + conventional drugs	Conventional drugs	2	RAS, ECG, CRP,

SPN: Sanqi Panax Notoginseng injection; RAS: reduction of angina symptoms; FAA: frequency of angina attack; DAA: duration of angina attack; GCRNDTCM: Guidelines of Clinical Research of New Drugs of Traditional Chinese Medicine; BL: blood lipid; CRP: C-reative protein.
